# Bidirectional association between thyroid autoimmunity/thyroglobulin antibody and diminished ovarian reserve: a systematic review and meta-analysis of observational studies

**DOI:** 10.3389/frph.2026.1958811

**Published:** 2026-10-20

**Authors:** Sini Yang, Jingjing Wang, Huawei Wang, Yuru Ma

**Affiliations:** 1 Reproductive Genetics Department, The First Affiliated Hospital of Kunming Medical University, Kunming, Yunnan, China; 2 Clinical Medical Research Center, The First Affiliated Hospital of Kunming Medical University, Kunming, Yunnan, China

**Keywords:** anti-Mullerian hormone, diminished ovarian reserve, premature ovarian insufficiency, thyroglobulin antibody, thyroid autoimmunity

## Abstract

**Background:**

Evidence suggests that thyroid autoimmunity (TAI) may be associated with ovarian dysfunction; however, most studies have focused primarily on thyroid peroxidase antibodies (TPOAb), while evidence regarding thyroglobulin antibodies (TgAb) remains limited and inconsistent. This study aimed to evaluate the association between TAI/TgAb and ovarian reserve.

**Methods:**

A systematic literature search was conducted in PubMed, Embase, Web of Science, CNKI databases for Chinese- and English-language articles published from January 2014 to January 2025. A bidirectional validation design was employed: (1) forward analysis, quantifying between-group differences in serum anti-Müllerian hormone (AMH) levels between TAI-positive women and healthy controls using standardized mean differences (SMD); and (2) reverse analysis, quantifying differences in TgAb levels (SMD for continuous variables) and TgAb positivity rates (odds ratios OR for dichotomous variables) between women with diminished ovarian reserve (DOR) and healthy controls. This study was prospectively registered with PROSPERO (CRD420251207428) and conducted in accordance with the PRISMA 2020 guidelines. The reverse analysis denotes a reverse-direction cross-sectional comparison in which the grouping and outcome variables are exchanged; it does not imply temporal or causal reversal.

**Results:**

Thirteen studies (total *n* = 4,286) were included. Forward analysis (4 studies, *n* = 870) demonstrated significantly lower serum AMH levels in TAI-positive women compared with controls (SMD=−0.20, 95% CI [−0.34, −0.06], *P* = 0.005, I^2^ = 0%). Reverse analysis of continuous variables (5 studies, *n* = 1,496) revealed significantly elevated TgAb levels in women with DOR compared with controls (SMD=0.93, 95% CI [0.36, 1.50], *P* = 0.001, I^2^ = 94%); however, this estimate should be interpreted with caution given the substantial heterogeneity. Reverse analysis of dichotomous variables (7 studies, *n* = 2,568) confirmed that women with DOR had significantly higher odds of TgAb positivity compared with controls (OR=1.65, 95% CI [1.29, 2.11], *P* < 0.0001, I^2^ = 32%).

**Conclusions:**

A significant bidirectional association exists between diminished ovarian reserve and TgAb/TAI positivity. TAI may impair ovarian reserve, while women with ovarian insufficiency exhibit a higher burden of thyroglobulin autoantibodies. These findings highlight the clinical importance of TgAb screening in the evaluation of ovarian function among reproductive-age women.

**Systematic Review Registration:**

https://www.crd.york.ac.uk/PROSPERO/view/CRD420251207428, identifier CRD420251207428.

## 1 Introduction

Premature ovarian insufficiency (POI) is a reproductive endocrine disorder characterized by the cessation of ovarian function before the age of 40 years and represents one of the leading causes of infertility in women of reproductive age (1). POI is not a discrete binary state but rather encompasses a continuum of ovarian dysfunction ranging from reduced fertility (occult POI) and elevated follicle-stimulating hormone (FSH) levels (biochemical POI) to amenorrhea (overt POI) (2). Epidemiological data indicate that the overall prevalence of POI among women of reproductive age is approximately 3.5%, with the prevalence of autoimmune-related POI reaching as high as 10.5% (3). To date, no effective strategies have been established to restore or preserve ovarian function and fertility in affected women (4), underscoring the critical clinical value of early identification of individuals at high risk of ovarian insufficiency. Anti-Müllerian hormone (AMH) has demonstrated clear advantages in assessing ovarian reserve and is regarded as the most accurate biomarker of ovarian reserve (5). AMH is produced by granulosa cells of primary, preantral, and small antral follicles and reflects the total number of antral and preantral follicles (6).

Thyroid autoimmunity (TAI), defined by the presence of anti-thyroid peroxidase antibodies (TPOAb), anti-thyroglobulin antibodies (TgAb), or both, is the most prevalent autoimmune condition among women of reproductive age (7). Notably, TAI is the autoimmune disease most closely associated with POI; the prevalence of TAI in women with POI (14–32.7%) is significantly higher than in the general reproductive-age population (1, 8), suggesting the existence of important pathophysiological links between the two conditions.

As two core serological markers of TAI, TPOAb and TgAb differ in clinical significance. Reports on the interaction between TgAb and AMH are limited and inconsistent: some studies have reported an inverse association between TgAb and AMH (9), whereas another reported a positive correlation (10), and still others found no significant relationship between the two (11, 12). These data implicate thyroid autoimmunity as a potential driver of accelerated ovarian aging between TgAb and AMH, along with the underlying mechanisms, remains to be elucidated.

Shared antigens between the thyroid and ovary may serve as potential targets for TAI-related autoantibodies in circulation or follicular fluid (13), providing a putative biological basis through which TAI may compromise ovarian function. Nevertheless, whether and how TAI affects ovarian reserve remains a subject of clinical debate. Existing meta-analyses have primarily focused on the association between TPOAb and ovarian reserve. Busnelli et al. (14) synthesized data from 30 studies and confirmed that Hashimoto's thyroiditis is significantly associated with reduced AMH levels and diminished antral follicle counts; however, systematic evidence specifically addressing TgAb remains extremely limited.

We employed a bidirectional validation design: a forward analysis to quantitatively assess differences in serum AMH levels between TAI-positive women and healthy controls, and a reverse analysis to quantitatively evaluate differences in TgAb levels and TgAb positivity rates between women with ovarian insufficiency and healthy controls. This meta-analysis aims to systematically integrate currently available scattered evidence and to systematically clarify the nature of the TgAb/TAI–DOR relationship between TgAb/TAI and ovarian reserve function, thereby providing evidence-based support for the clinical utility of TgAb screening in the assessment of ovarian function in women of reproductive age.

## 2 Methods

### 2.1 Literature search

PubMed, Embase, Web of Science, CNKI databases were systematically searched for eligible studies published from January 1, 2014 to January 1, 2025. The search strategy combined Medical Subject Headings (MeSH terms) with free-text terms, including: thyroid autoimmunity, Hashimoto's thyroiditis, thyroglobulin antibody, TgAb, ovarian reserve, anti-Müllerian hormone, AMH, diminished ovarian reserve, premature ovarian insufficiency, and their Chinese-language equivalents. The complete search strategies for each database are provided in Supplementary Figure S1. All retrieved articles were independently screened by two reviewers, and reference lists of eligible studies were manually searched for additional relevant publications. Disagreements were resolved by discussion or by consultation with a third reviewer.

### 2.2 Eligibility criteria

Eligibility was prospectively specified using the PICOS framework: Population—women of reproductive age (18–45 years); Intervention/Exposure—thyroid autoimmunity (thyroglobulin antibody TgAb and/or thyroid peroxidase antibody TPOAb positivity, or a diagnosis of autoimmune thyroid disease); Comparator—women without thyroid autoimmunity for the forward analysis, or women without diminished ovarian reserve for the reverse-direction analysis; Outcome—ovarian reserve measured by serum anti-Müllerian hormone (AMH) as the primary marker, with antral follicle count and basal follicle-stimulating hormone as secondary markers where reported, for the forward analysis, and serum TgAb levels and TgAb positivity rates for the reverse-direction analysis; Study design—observational studies (cross-sectional, case–control, or cohort).

Studies were included if they met all of the following criteria: (1) participants were women of reproductive age (18–45 years); (2) ovarian reserve was assessed using at least one validated marker, including serum AMH, basal FSH, or transvaginal AFC; (3) participants were assessed for thyroid autoimmunity (diagnosed with autoimmune thyroid disease AITD or with TgAb and/or TPOAb measurement results available), including euthyroid women with subclinical TAI (TAI-positive without overt thyroid dysfunction); and (4) the study design was observational (cross-sectional, case-control, or cohort).

Studies were excluded for the following reasons: (1) non-human studies, case reports, reviews, conference abstracts, or articles in languages other than Chinese or English; (2) participants had a history of thyroid-directed treatment (antithyroid drugs, radioactive iodine, or thyroid surgery); (3) participants had other autoimmune diseases, iatrogenic ovarian injury, chemotherapy, radiotherapy, or a diagnosis of polycystic ovary syndrome(PCOS); (4) chromosomal abnormalities or congenital ovarian dysgenesis.

Consistent with this framework, women with PCOS were excluded, and for studies enrolling mixed populations only the non-PCOS subgroup was eligible. This decision was made *a priori* because PCOS is independently associated with elevated AMH owing to increased preantral and small antral follicle numbers, which would confound the TAI/TgAb-ovarian reserve association.

Bidirectional analytical design. Two complementary observational comparisons were performed. The forward analysis compared serum AMH between TAI-positive (or TgAb-positive) women and healthy controls, addressing whether thyroid autoimmunity is associated with lower ovarian reserve. The reverse-direction analysis compared serum TgAb levels and TgAb positivity rates between women with diminished ovarian reserve (DOR) and healthy controls, addressing the complementary question of whether ovarian insufficiency is associated with a greater burden of thyroglobulin autoimmunity. The term ‘reverse-direction’ is descriptive: it indicates that the grouping and outcome variables are exchanged relative to the forward analysis, and it does not imply a temporal reversal, a reversal of causation, or a longitudinal design. Because all included studies are observational (predominantly cross-sectional), neither direction establishes temporality or causality; the reverse-direction comparison is therefore hypothesis-generating and complementary rather than confirmatory.

### 2.3 Data extraction

Two reviewers independently extracted the following data: first author, publication year, study design, participant characteristics, sample size, and laboratory data including serum TSH, FT3, FT4, TPOAb, TgAb, AMH, and AFC. When AMH or TgAb levels were reported as median and range (or interquartile range) rather than mean and standard deviation, values were converted using the method of Luo et al. (15) implemented in RStudio.

Methodological quality was assessed using the Newcastle-Ottawa Scale (NOS; range 0–9 points); scores ≥6 were considered acceptable. NOS scores are provided in Supplementary Table S1.

### 2.4 Statistical analysis

Between-group differences in serum AMH levels and TgAb levels were quantified using SMD with 95% confidence intervals (CI). Between-group differences in TgAb positivity rates were expressed as OR with 95% CI. Statistical significance was defined as *P* < 0.05. Heterogeneity was assessed using the chi-squared test and quantified using the I^2^ statistic; values of 25%, 50%, and 75% were considered low, moderate, and high heterogeneity, respectively (16). A random-effects model (DerSimonian–Laird) was applied when I^2^ > 50%; otherwise, a fixed-effects model was used. For analyses with substantial heterogeneity, Galbraith plots were constructed, *τ*^2^ and prediction intervals were reported, and *post-hoc* subgroup analyses were conducted to explore sources of heterogeneity.

Due to fewer than 10 studies per analysis, funnel plots and Egger's/Begg's tests lacked sufficient power for reliable publication bias detection (17); formal quantitative assessment of publication bias was therefore not performed. This is an acknowledged limitation. To minimize potential publication bias, systematic multi-database searching and manual reference checking were employed.

## 3 Results

### 3.1 Study selection

The initial database search identified records from five databases; following deduplication, eligible records were screened by title and abstract. After title and abstract screening, 18 studies proceeded to full-text assessment, of which 5 were excluded for not meeting inclusion criteria. Two studies (18, 19) were highly relevant but could not be included in quantitative synthesis due to absence of extractable effect-size data; these were retained for qualitative analysis. Thirteen studies were ultimately included. The PRISMA 2020 flow diagram is shown in Figure 1.

**Figure 1:**
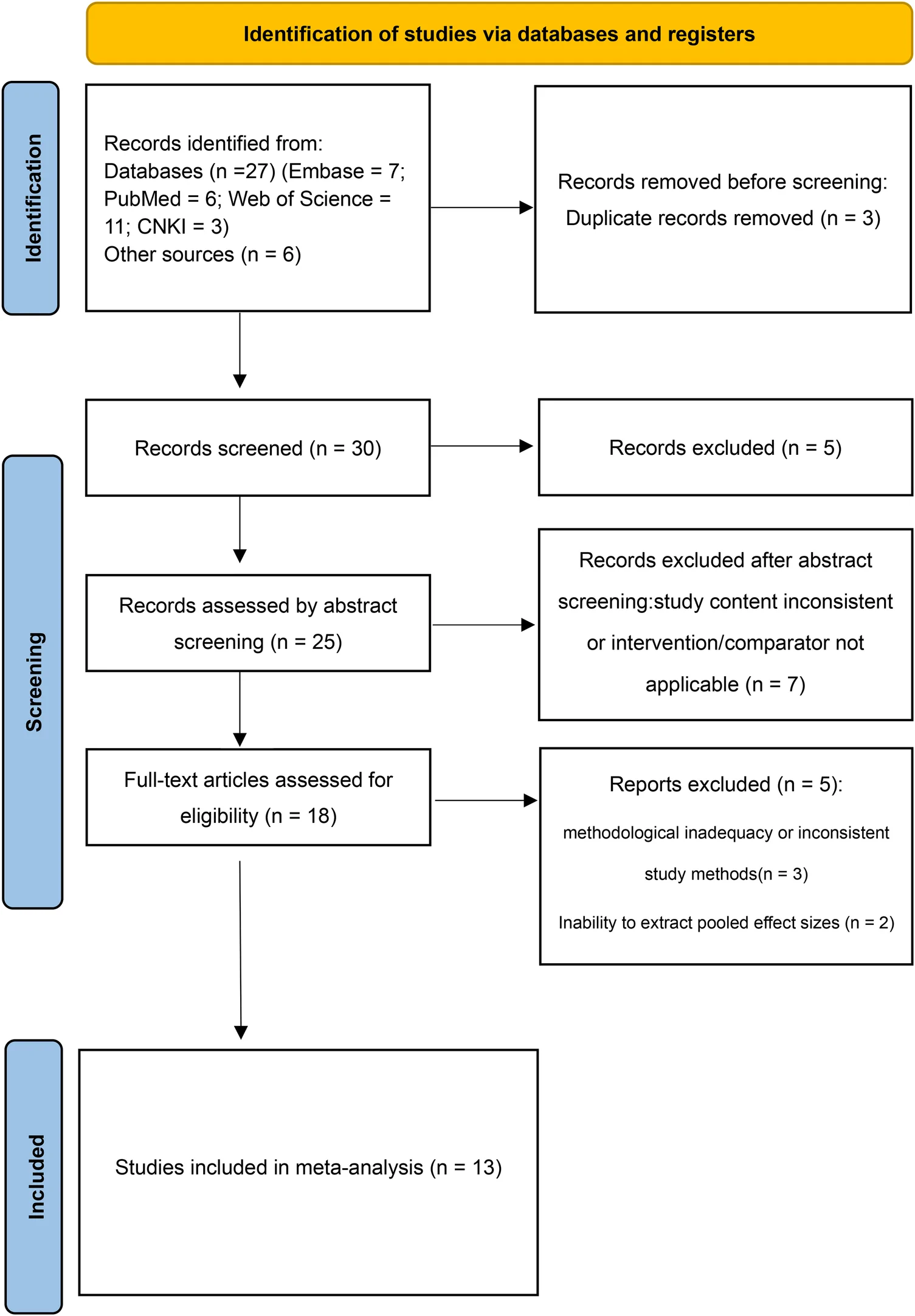
PRISMA 2020 flow diagram of the search process.

Two studies published in 2022 by the same first author were both included; after independent evaluation by two reviewers, these were deemed to represent independent research (different study populations, data sources, and primary outcomes, published in distinct journals) with no evidence of overlapping participants.

### 3.2 Characteristics of included studies

Characteristics of included studies are summarized in Table 1. Of the 13 included studies, 8 were cross-sectional, 3 were case-control, and 2 were cohort studies. Publication years ranged from 2015 to 2025; sample sizes ranged from 52 to 1,044, with a total of 4,286 participants. All studies enrolled reproductive-age women (predominantly 20–40 years), including healthy controls, infertile women, women with AITD, and women with impaired ovarian reserve (LOR, POI, and premature menopause). All studies assessed ovarian reserve markers and thyroid-related indices. Methodological quality was generally satisfactory (NOS scores: 6–9).

**Table 1:** Basic characteristics of the included studies.

Author	Year	Country	Study design	Study population	Sample size and classification	Thyroid function tests and antibodies	Ovarian reserve assessment	Key findings	Quality assessment (NOS score)
Felipe A. Morales-Martínez	2021	Mexico	Prospective cohort study	Women of reproductive age (20–35 years) with primary autoimmune hypothyroidism	*n* = 52 Thyroid autoimmune disease group (TAID): *n* = 27 Control group: *n* = 25	TSH, FT4, TPOAb	AMH, AFC, ovarian volume	No significant differences between groups in menstrual cycle length, serum AMH levels, or AFC	9
Li-li Zhuang	2017	China	Prospective cohort study	Euthyroid infertile women undergoing IVF-ET treatment	*n* = 350 ATA-positive group: *n* = 140 ATA-negative group: *n* = 210	TSH, FT3, FT4, TPOAb, TgAb	AMH, number of oocytes retrieved	AMH levels were slightly lower in the ATA-positive group than in the ATA-negative group, but the difference was not statistically significant (*p* > 0.05)	9
İlknur Öztürk Ünsal	2021	Turkey	Retrospective case-control study	Euthyroid women of reproductive age (18–40 years) diagnosed with Hashimoto’s thyroiditis	*n* = 280 Hashimoto's thyroiditis group (HT): *n* = 108 Control group: *n* = 172	TSH, FT4, TPOAb, TgAb	AMH	Serum AMH levels were significantly lower in the HT group than in controls. Ovarian reserve assessed by AMH was affected by thyroid autoimmunity regardless of antibody type or titer	9
Adriana Leal Griz Notaro	2024	Brazil	Retrospective cross-sectional study	Women of reproductive age (18–47 years) attending an assisted reproduction clinic in Brazil	*n* = 188 Thyroid autoimmune group (TAI): *n* = 63 Control group: *n* = 125	TSH, FT4, TPOAb, TgAb	AMH, AFC	No difference in median AMH or AFC between groups; however, in a subgroup analysis by age, TAI women aged > 39 years showed a trend toward lower median AMH (0.9 ng/mL vs. 1.5 ng/mL, *p* = 0.08)	9
Nearmeen M. Rashad	2018	Egypt	Cross-sectional study	Egyptian women with autoimmune thyroiditis	*n* = 200 Premature menopause group: *n* = 54 Natural menopause group: *n* = 69	TSH, FT3, FT4, TPOAb, TgAb	AMH, AFC, ovarian volume	Linear regression analysis showed that AMH levels were independently associated with age, anti-TG antibody, and ovarian volume; subclinical hypothyroidism (SCHT) showed the highest prevalence	9
K. Beitl, J. Ott	2024	Austria	Prospective case-control study	Patients with spontaneous POI attending the Clinical Division of Reproductive Medicine, Medical University of Vienna, May 2022—June 2023	*n* = 132 Spontaneous POI (excluding chromosomal abnormalities and iatrogenic causes): *n* = 66 Healthy controls: *n* = 66	TSH, TPOAb, TgAb	AMH	Women with POI showed significantly elevated TgAb (*p* = 0.045) and TPOAb (*p* = 0.002) compared with controls	8
Jazyra Zynat	2023	China	Retrospective cross-sectional study	Women aged 18–45 years from two communities in Ürümqi, Xinjiang, and attending the People’s Hospital of Xinjiang Uyghur Autonomous Region (January 2016—April 2019)	*n* = 483 Low-AMH group: *n* = 48 Normal-AMH group: *n* = 388	TSH, FT3, FT4, TPOAb, TgAb	AMH	TgAb was significantly associated with low AMH; each unit increase in TgAb increased the odds of AMH below the 10th percentile by 1.012-fold (*p* = 0.032). TSH, TPOAb, and age were not significantly associated with low AMH (*p* > 0.05)	8
Zhang Shuo (Thesis)	2022	China	Retrospective cross-sectional study	Female patients attending the Reproductive Hospital Affiliated to Shandong University for their first assisted reproduction treatment (May 2014—January 2021)	*n* = 386 Low ovarian reserve (LOR) group: *n* = 116 Normal ovarian reserve (NOR) group: *n* = 270	TSH, FT3, FT4, TPOAb, TgAb	AMH	No statistically significant differences between groups in TSH, FT4, FT3, TPOAb, TgAb, prevalence of hypothyroidism, anti-thyroid drug (ATD) use, or positive anti-thyroid antibodies (all *p* > 0.05). NOR patients were older (*p* = 0.04) and had higher BMI (*p* = 0.038)	9
Zhang Shuo	2022	China	Retrospective cross-sectional study	Patients attending the Reproductive Hospital Affiliated to Shandong University for their first assisted reproduction treatment (May 2014—January 2021)	*n* = 348 LOR group: *n* = 64 NOR group: *n* = 284	TSH, FT3, FT4, TPOAb, TgAb	AMH	No statistically significant differences between groups in BMI, TSH, FT3, FT4, TPOAb, or TgAb (all *p* > 0.05). The DOR group was significantly older than the NOR group (*p* < 0.05)	9
Guo Yan	2023	China	Retrospective cross-sectional study	Female outpatients aged 20–40 years attending the Department of Reproductive Medicine, First Affiliated Hospital of Shenzhen University (January 2021—October 2022)	*n* = 229 LOR group: *n* = 35 NOR group: *n* = 168	TSH, FT3, FT4, TPOAb, TgAb	AMH	TPOAb was significantly negatively correlated with AMH (Pearson r = −0.252, *p* < 0.01); TgAb was also significantly negatively correlated with AMH (Pearson r = −0.203, *p* < 0.01), suggesting that anti-thyroid antibodies may impair ovarian reserve function	9
Jie Wu	2021	China	Retrospective cross-sectional study	Infertile women attending the Affiliated Hospital of Southwest Medical University and Fushun Maternal and Child Health Hospital (January 2019—December 2020)	*n* = 496 (mean age 30.31 ± 4.50 years); subgrouped by TSH level, thyroid antibodies, age, AMH, FSH, and other criteria	TSH, FT3, FT4, TPOAb, TgAb	AMH, AFC	No statistically significant differences among groups in age, AMH, or FSH (*p* > 0.05); no significant differences in TSH, FT3, or FT4 (*p* > 0.05)	6
Andrea Weghofer	2015	USA	Case-control study	Infertile women undergoing their first IVF cycle at a reproductive center in New York City (January 2011—February 2013)	*n* = 98 TSH ≤ 2.5 *μ*IU/mL group: *n* = 77 TSH > 2.5 μIU/mL group: *n* = 21	TSH, TPOAb, TgAb	AMH	TPO antibodies significantly affected embryo quality in women with TSH below the normal range (*p* = 0.045). In women with TSH above normal, higher TSH had a negative impact on embryo quality (*p* = 0.027)	7
Chien-Wen Chen	2017	Taiwan, China	Cross-sectional study	Infertile women attending Taipei Medical University-affiliated hospitals (October 2013—March 2016)	*n* = 1,044 Low ovarian reserve group: *n* = 157 Normal ovarian reserve group: *n* = 729 High ovarian reserve group: *n* = 158	TSH, TPOAb, TgAb	AMH	In Chinese women, idiopathic diminished ovarian reserve was associated with positive TPOAb, but not with thyroid function status or positive TgAb	8

AMH, anti-Müllerian hormone; AFC, antral follicle count; TSH, thyroid-stimulating hormone; FT3, free triiodothyronine; FT4, free thyroxine; TPOAb, anti-thyroid peroxidase antibody; TgAb, anti-thyroglobulin antibody; ATA, anti-thyroid antibody; IVF-ET, *in vitro* fertilization and embryo transfer; HT, Hashimoto’s thyroiditis; POI, premature ovarian insufficiency; LOR, low ovarian reserve; NOR, normal ovarian reserve; DOR, diminished ovarian reserve; NOS, Newcastle-Ottawa scale.

### 3.3 Meta-analysis results

#### 3.3.1 Forward analysis: TAI and serum AMH levels

Four studies compared AMH levels between euthyroid TAI-positive women and healthy controls. Pooled analysis revealed significantly lower AMH levels in TAI-positive women (SMD = −0.20, 95% CI: −0.34 to −0.06, *P* = 0.005, I^2^ = 0%), indicating a statistically significant but modest reduction in ovarian reserve with low heterogeneity (Figure 2).

**Figure 2:**
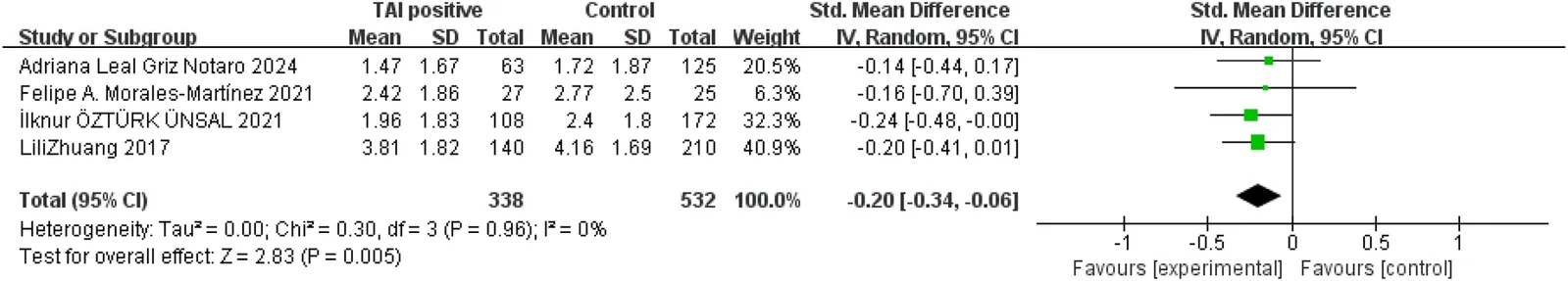
Forest plot of the difference in serum AMH levels between women with thyroid autoimmunity (TAI) and the control group (SMD). AMH, anti-Müllerian hormone.

#### 3.3.2 Reverse analysis: serum TgAb levels in women with DOR

Five studies were included in this analysis. The spectrum of ovarian insufficiency ranged from subclinical DOR to confirmed POI and premature menopause. Women with LOR had significantly elevated TgAb levels compared with controls (SMD = 0.93, 95% CI: 0.36 to 1.50, *P* = 0.001, I^2^ = 94%, *P* < 0.00001).

A Galbraith plot identified three studies as major contributors to heterogeneity (Figure 3): Jazyra Zynat 2023 (Xinjiang, China), Nearmeen M. Rashad 2018 (Egypt), and GuoYan 2023 (the Greater Bay Area of China), all of which fell outside the ±1.96 reference bands. The 95% prediction interval (−1.26 to 3.12), which crosses zero, indicates that the true effect size is likely to vary considerably across populations, and that the association may be negligible or absent in some settings. Given the high I^2^ value and wide prediction interval, this pooled estimate should be interpreted with caution (Figure 4).

**Figure 3:**
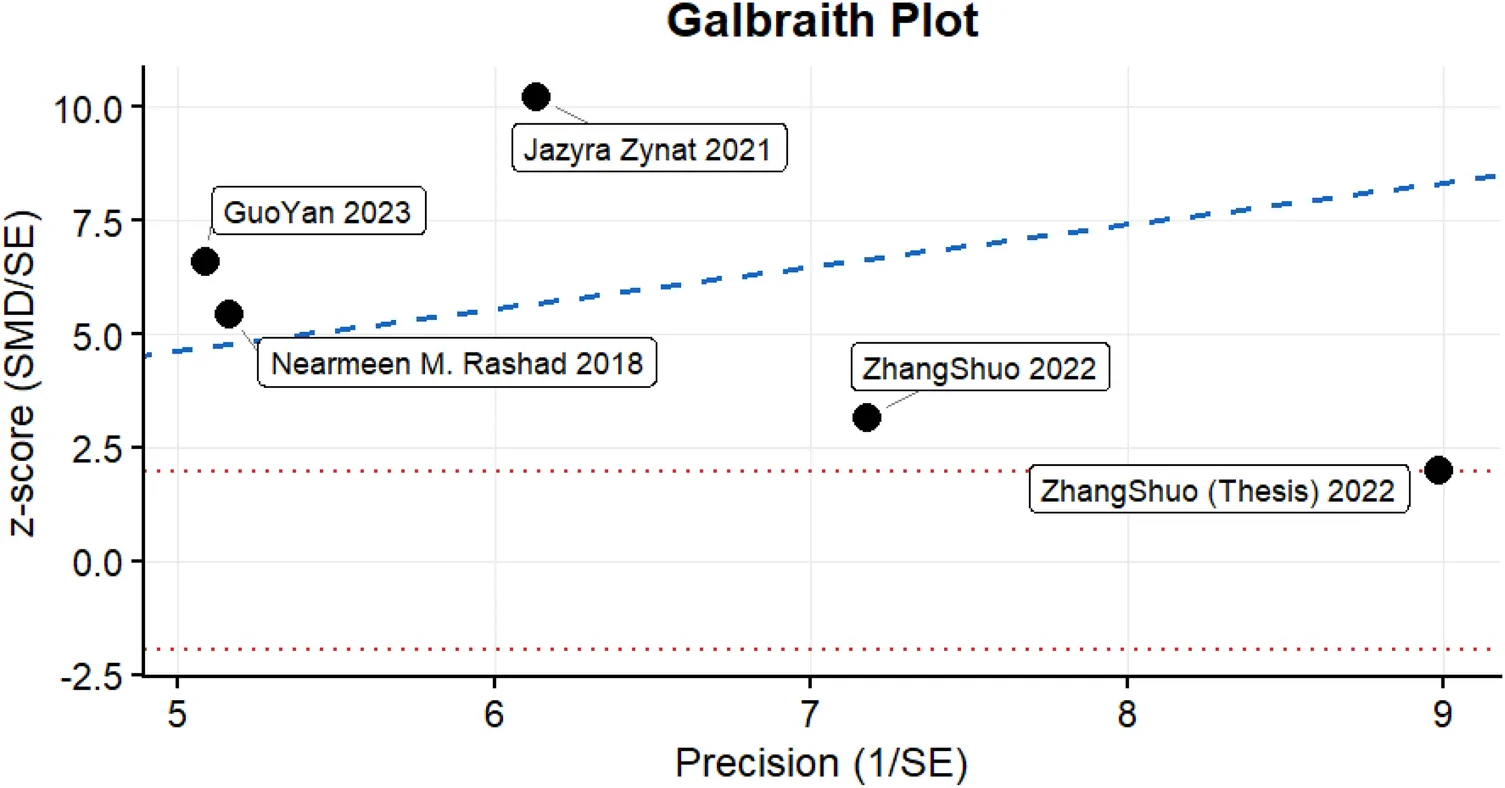
Galbraith plot illustrating heterogeneity contributors in the reverse continuous-variable analysis.

**Figure 4:**
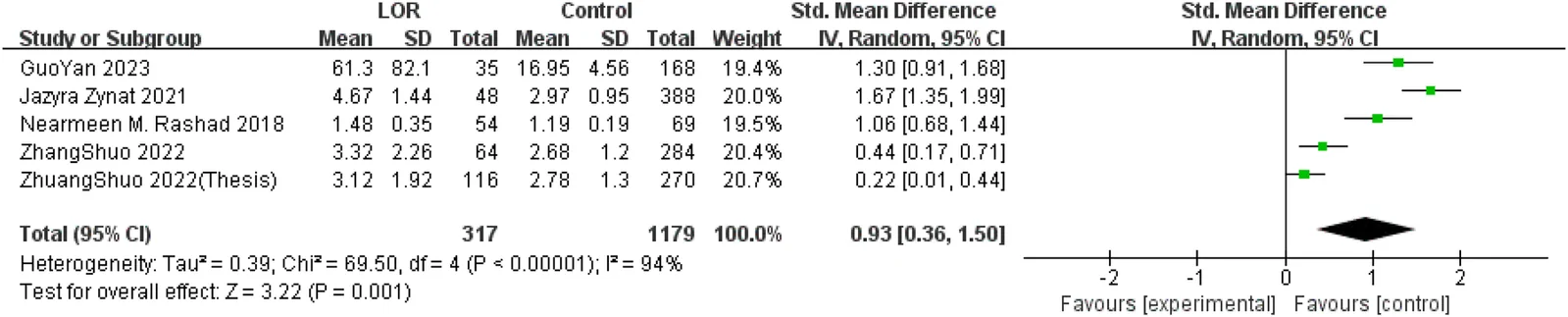
Forest plot of the difference in serum TgAb levels between women with low ovarian reserve and the control group (SMD). TgAb, anti-thyroglobulin antibody.

#### 3.3.3 Reverse analysis: TgAb positivity rate in women with DOR

Seven studies were included. Women with DOR had significantly higher odds of TgAb positivity compared with healthy controls (OR=1.65, 95% CI: 1.29 to 2.11, *P* < 0.0001, I^2^ = 32%), with low-to-moderate heterogeneity supporting the consistency and robustness of this estimate (Figure 5).

**Figure 5:**
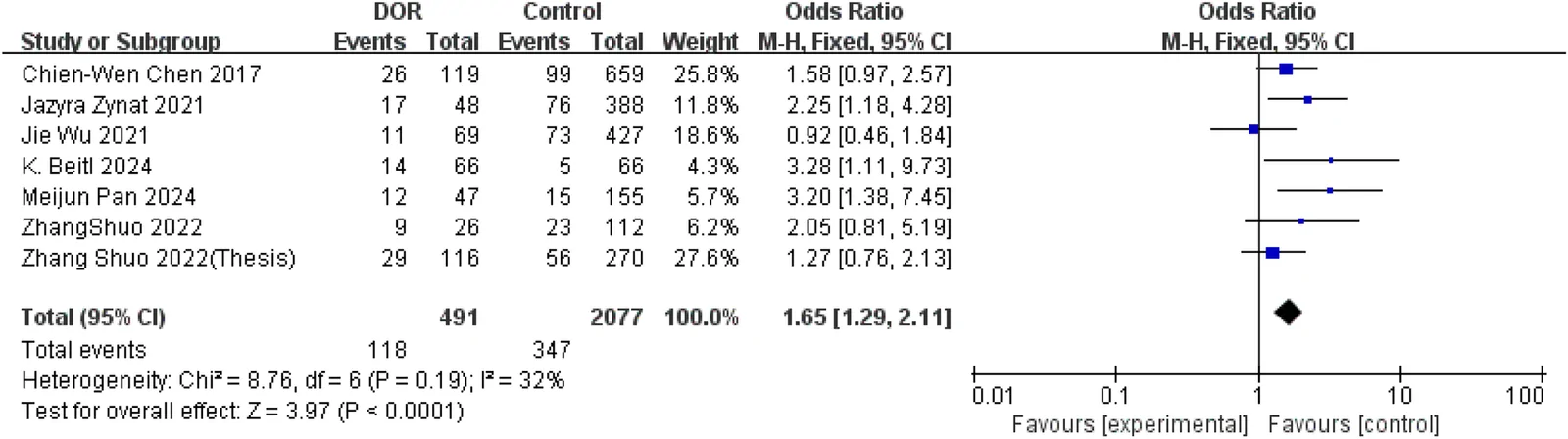
Forest plot (OR) showing the difference in TgAb positivity rate between women with diminished ovarian reserve and the control group. TgAb, anti-thyroglobulin antibody.

### 3.4 Sensitivity analysis

Given the small number of included studies (*n* = 4–7 per analysis), leave-one-out sensitivity analysis has limited statistical validity (20). We therefore performed a qualitative external consistency assessment using independent studies not included in the primary meta-analysis.

For the forward analysis (TAI → reduced AMH; SMD = −0.20, I^2^ = 0%), two large independent studies are corroborative: Busnelli et al. (14) confirmed in an updated systematic review of 30 studies that Hashimoto's thyroiditis is significantly associated with reduced AMH (SMD = −0.35, 95% CI: −0.51 to −0.19, *P* < 0.0001) and decreased AFC; Hsieh and Ho (21) found in a nationwide database study (*n* = 21,325) that TAI significantly increased POI risk (aOR = 1.36, 95% CI: 1.12–1.65).

For the reverse analysis (DOR → elevated TgAb; OR=1.65, I^2^ = 32%), Li et al. (22) in a systematic review of 14 studies found that TAI prevalence (including TgAb positivity) was significantly higher in women with DOR/POI than in controls (OR=2.07, 95% CI: 1.61–2.67), corroborating the reverse association.

Two studies not included in quantitative synthesis provided complementary evidence: Zhang et al. (18) found that subclinical/overt hypothyroidism—but not TAI positivity alone—was significantly associated with DOR risk (OR=1.67, 95% CI: 1.08–2.57), suggesting that thyroid function status may independently influence DOR, and that thyroid function should be considered as a confounder when interpreting the forward analysis results. Pan et al. (19) reported that isolated TgAb elevation did not affect ovarian reserve in a murine autoimmune thyroiditis model, suggesting TgAb may function primarily as a marker of systemic immune activation rather than a direct pathogenic effector.

## 4 Discussion

Diminished ovarian reserve is a hallmark of female reproductive aging; however, the present findings indicate that a subset of reproductive-age women who are not yet elderly already exhibit significant DOR, suggesting that immune factors may contribute to premature ovarian aging beyond the influence of chronological age (23, 24). The prevalence of TPOAb and TgAb positivity among reproductive-age women is approximately 8%–14%, making TAI the most prevalent autoimmune condition in this demographic (25). Given that premature ovarian insufficiency (POI) represents a stage within a continuum of progressive ovarian decline (1, 26), the present findings further indicating that thyroid autoimmunity may play a contributory role in this pathological progression.

Mechanistically, the association between thyroid autoimmunity and ovarian dysfunction may be explained at multiple biological levels. First, at the local ovarian level, circulating thyroid autoantibodies may penetrate the blood–follicle barrier and directly interact with granulosa cells, as supported by their detection in follicular fluid (27). Second, at the cellular level, immune-driven generation of reactive oxygen species (ROS) and activation of Fas/FasL signaling are established drivers of granulosa cell apoptosis and follicular atresia (28, 29). Triiodothyronine (T3) normally confers cytoprotection to granulosa cells via the PI3 K/Akt pathway (30); TAI-mediated attenuation of local thyroid hormone signaling therefore removes this protective mechanism. Third, at the systemic level, T-cell subset imbalances and dysregulated pro-inflammatory cytokines—including IL-6 and TNF-α—may further destabilize the intraovarian immune milieu (31). Notably, even moderately elevated TSH concentrations within the normal reference range have been inversely associated with ovarian reserve markers (32), suggesting that functional cross-talk between the hypothalamic-pituitary-thyroid and hypothalamic-pituitary-ovarian axes may amplify immune-mediated follicular depletion. These converging mechanistic lines provide a coherent biological framework for the association examined in the present meta-analysis.

An additional consideration relevant to the infertility setting is polycystic ovary syndrome (PCOS). Because PCOS is characterised by intrinsically elevated AMH owing to an increased preantral and small antral follicle pool, and because autoimmune thyroiditis and PCOS frequently coexist, women with PCOS were deliberately excluded from the present analyses. Palomba et al. (33) recently proposed that, during its initial phases, autoimmune thyroiditis may induce an inflammatory process that also involves the ovary and may predispose young women to PCOS or to a more severe PCOS phenotype; in this early phase, thyroid autoimmunity may be associated with higher, rather than lower, AMH, whereas chronic, long-standing autoimmune inflammation is expected to deplete the follicular pool and ultimately reduce ovarian reserve.

This biphasic, time-dependent model has two implications for interpreting our results. First, the included studies reported a single, cross-sectional assessment of AMH and thyroid antibodies and generally did not report the duration of thyroid autoimmunity before ovarian reserve assessment; the absence of data on the initial (early-phase) effect of autoimmunity and on autoimmune exposure duration is therefore a potential confounder, because the direction and magnitude of the observed TAI/TgAb-AMH association depend on when in the disease course women were assessed. Second, our forward analysis captured a modest reduction in AMH (SMD = −0.20), which is consistent with a population assessed, on average, after the early inflammatory phase; studies that include PCOS women or sample shortly after autoimmunity onset may observe null or even positive associations. Future studies should report autoimmune exposure duration, serially measure AMH and thyroid antibodies, and, even when PCOS patients are the focus, include a non-PCOS comparator group so that the disease-specific and autoimmune-specific contributions to ovarian reserve can be disentangled.

In the forward analytical direction, the present meta-analysis demonstrated that euthyroid women with TAI had significantly lower serum AMH levels than healthy controls, indicating a modest but statistically robust reduction in ovarian reserve. Crucially, this finding was obtained in euthyroid women, isolating the autoimmune component from the confounding influence of overt thyroid dysfunction and supporting the hypothesis that immune dysregulation *per se* may contribute to impaired follicular development. This conclusion is consistent with and corroborated by two large independent bodies of evidence. An updated meta-analysis by Busnelli et al. (14), synthesising data from 30 studies, confirmed that Hashimoto's thyroiditis is significantly associated with reduced AMH and decreased antral follicle count (AFC). A nationwide database cohort study by Hsieh and Ho (21) (*n* = 21,325) further demonstrated that TAI independently increased the risk of premature ovarian insufficiency. The convergence of the present result with these external data substantially strengthens confidence in the directionality and magnitude of the forward association.

Busnelli et al. (14) represents the most comprehensive synthesis of thyroid autoimmunity and ovarian reserve to date, but its design carries several limitations that our study was intended to address. First, that meta-analysis assessed thyroid autoimmunity broadly, driven predominantly by TPOAb and Hashimoto's thyroiditis and did not provide a dedicated, separate pooled estimate for TgAb; the present study isolates TgAb as the exposure/outcome of interest and, in parallel, retains a TAI-level forward analysis. Second, it pooled euthyroid, subclinically hypothyroid, and assisted-reproduction populations with inconsistent adjustment for thyroid function, which may confound the autoimmune effect with thyroid dysfunction; we restricted the forward analysis to euthyroid TAI-positive women to isolate the autoimmune component. Our results therefore complement, rather than duplicate, the findings of Busnelli et al.

Nevertheless, inconsistencies remain across individual studies. Korevaar et al. (34) reported a positive association between TgAb positivity and AFC after multivariable adjustment, and Chen et al. (35) found no significant difference in ovarian reserve markers by TgAb status. These discrepancies are likely attributable to methodological heterogeneity, including variation in TgAb positivity thresholds (e.g., >25 IU/mL vs. >115 IU/mL), differences in assay platforms, and population-specific genetic and environmental factors (36). By pooling available evidence through meta-analysis, the present study offers a more integrated and statistically robust estimate than any single investigation.

The reverse analytical direction yielded complementary and conceptually important findings. Women with DOR exhibited significantly elevated serum TgAb levels and significantly higher odds of TgAb positivity compared with healthy controls, indicating that diminished ovarian reserve is accompanied by a measurably greater burden of thyroglobulin autoimmunity. These observations are closely aligned with a systematic review by Li et al. (22), which reported that TAI prevalence—including TgAb positivity—was significantly higher in women with DOR/POI than in controls. Collectively, these findings suggest that ovarian reserve decline is not merely a downstream consequence of immune injury but may itself be associated with, or promote, systemic immune activation—possibly through endocrine feedback mechanisms linking progressive loss of ovarian endocrine function to dysregulation of immune homeostasis. Whether this represents bidirectional causality or a shared immunogenetic diathesis warrants investigation in well-designed prospective cohort studies.

The high statistical heterogeneity (I^2^ = 94%) observed in the reverse continuous-variable analysis warrants explicit acknowledgement. Galbraith plot analysis identified three major contributors—Jazyra Zynat 2023 (Xinjiang, China), GuoYan 2023 (Greater Bay Area, China), and Nearmeen M. Rashad 2018 (Egypt) — likely attributable to clinically meaningful differences in ethnic background, iodine status, assay methodology. The wide prediction interval crossing zero further indicates that the true effect size is likely to vary substantially across populations, and the pooled estimate from this analysis should therefore be interpreted with appropriate caution. In contrast, the reverse dichotomous analysis (OR=1.65; I^2^ = 32%) was characterised by low-to-moderate heterogeneity, providing a more consistent and reliable summary estimate that is robust across the included study populations.

It is also important to note that two studies not eligible for quantitative synthesis provided complementary mechanistic insights. Zhang et al. (18) found that subclinical and overt hypothyroidism—but not isolated TAI positivity—was independently associated with DOR risk, highlighting thyroid function status as a potential independent confounder that future studies should systematically address. Pan et al. (19) reported that isolated TgAb elevation did not impair ovarian reserve in a murine autoimmune thyroiditis model, suggesting that TgAb may function primarily as a marker of broader immune activation rather than a direct pathogenic effector. These observations do not negate the epidemiological associations demonstrated here, but they do underscore the complexity of the TgAb–DOR relationship and the need for mechanistic studies to delineate direct from indirect pathways.

A principal strength of the present study is the novel bidirectional meta-analysis design, which—to our knowledge—has not previously been applied to this research question. Simultaneous forward and reverse analyses validate the TgAb/TAI–DOR association from two independent analytical directions, substantially enhancing the internal credibility of the conclusions. The quantitative findings were further corroborated by high-quality external evidence, including a nationwide cohort study (21) and an independent systematic review (22), neither of which was included in the primary meta-analyses.

Several limitations should be acknowledged. First, adjustment for key confounders (e.g., BMI, age, TSH within the normal range, and coexisting TPOAb positivity) was inconsistent across studies. Second, the included evidence base is predominantly derived from East Asian populations; established inter-ethnic differences in TAI prevalence, TgAb positivity thresholds, and ovarian aging trajectories limit generalisability to other ethnic groups. Third, the small number of studies per analysis (*n* = 4–7) precluded reliable funnel-plot-based assessment of publication bias; this limitation was partially mitigated by systematic multi-database searching and manual reference checking. Future studies integrating immunological profiling and longitudinal ovarian reserve assessment are needed to clarify causality and identify potential therapeutic targets.

It is important to emphasise that the present findings do not imply that elevated TgAb is invariably a direct causative factor in ovarian reserve decline. In several individual studies, TgAb positivity was not significantly associated with ovarian reserve markers (27, 37, 38), suggesting that TgAb may function more as a marker of systemic immune activation than as an independent pathogenic effector. The relationship between TgAb and ovarian function may be modulated by TSH levels, TAI duration, antibody titres, and genetic background.

## 5 Conclusions

This study demonstrates a significant bidirectional association between diminished ovarian reserve and TgAb/TAI positivity, providing novel epidemiological evidence that autoimmune mechanisms participate in the pathological process of ovarian functional decline. These findings suggest that TgAb screening may be considered in the evaluation of ovarian reserve in reproductive-age women with unexplained infertility or DOR, even in the setting of normal thyroid function. Prospective longitudinal monitoring of ovarian reserve markers (AMH, AFC) in TAI-positive women may facilitate early identification of individuals at risk for accelerated ovarian aging and inform timely fertility preservation counselling.

## Data Availability

The original contributions presented in the study are included in the article/Supplementary Material, further inquiries can be directed to the corresponding author.
