# Supplementary material for: Bidirectional association between thyroid autoimmunity/thyroglobulin antibody and diminished ovarian reserve: a systematic review and meta-analysis of observational studies
Source: Front Reprod Health. 2026 Oct 20;8:1958811. doi: 10.3389/frph.2026.1958811 (PMC13727643; doi:10.3389/frph.2026.1958811)
Supplement: Supplementary file 1 [file Datasheet1.docx]

**Supplementary Figure S1. Literature Search Strategy**

1. PubMed Search Strategy

Search period: January 1, 2014 – January 1, 2025

#1 "Thyroid Diseases"[MeSH] OR "Thyroiditis, Autoimmune"[MeSH] OR "Hashimoto Disease"[MeSH]

#2 "thyroid autoimmunity"[tiab] OR "autoimmune thyroid"[tiab] OR

"Hashimoto thyroiditis"[tiab] OR "Hashimoto's thyroiditis"[tiab] OR

"autoimmune thyroiditis"[tiab]

#3 "thyroglobulin antibod*"[tiab] OR "anti-thyroglobulin antibod*"[tiab] OR

"TgAb"[tiab] OR "TGAb"[tiab] OR "anti-Tg"[tiab]

#4 "thyroid peroxidase antibod*"[tiab] OR "TPOAb"[tiab] OR

"anti-TPO"[tiab] OR "antithyroid antibod*"[tiab]

#5 #1 OR #2 OR #3 OR #4

#6 "Ovarian Reserve"[MeSH] OR "Ovarian Insufficiency, Primary"[MeSH] OR

"Menopause, Premature"[MeSH]

#7 "ovarian reserve"[tiab] OR "diminished ovarian reserve"[tiab] OR

"reduced ovarian reserve"[tiab] OR "poor ovarian reserve"[tiab]

#8 "premature ovarian insufficiency"[tiab] OR

"premature ovarian failure"[tiab] OR "POI"[tiab] OR "POF"[tiab]

#9 "anti-Mullerian hormone"[tiab] OR "AMH"[tiab] OR

"Mullerian inhibiting substance"[tiab]

#10 "antral follicle count"[tiab] OR "AFC"[tiab] OR

"follicle stimulating hormone"[tiab] OR "FSH"[tiab]

#11 #6 OR #7 OR #8 OR #9 OR #10

#12 #5 AND #11

#13 "humans"[MeSH] AND ("female"[MeSH] OR "women"[tiab])

#14 #12 AND #13

**Filters applied:**

Publication date: January 1, 2014 – January 1, 2025 Language: English and Chinese

**Final Search Query (Single-Line Format)：**

(("Thyroid Diseases"[MeSH] OR "Thyroiditis, Autoimmune"[MeSH] OR

"Hashimoto Disease"[MeSH] OR "thyroid autoimmunity"[tiab] OR

"autoimmune thyroid"[tiab] OR "Hashimoto thyroiditis"[tiab] OR

"thyroglobulin antibod*"[tiab] OR "TgAb"[tiab] OR "TGAb"[tiab] OR

"anti-Tg"[tiab] OR "thyroid peroxidase antibod*"[tiab] OR

"TPOAb"[tiab] OR "antithyroid antibod*"[tiab])

AND

("Ovarian Reserve"[MeSH] OR "Ovarian Insufficiency, Primary"[MeSH] OR

"ovarian reserve"[tiab] OR "diminished ovarian reserve"[tiab] OR

"premature ovarian insufficiency"[tiab] OR

"premature ovarian failure"[tiab] OR "POI"[tiab] OR

"anti-Mullerian hormone"[tiab] OR "AMH"[tiab] OR

"antral follicle count"[tiab] OR "AFC"[tiab])

AND

("humans"[MeSH]) AND ("female"[MeSH] OR "women"[tiab]))

### **2. Embase Search Strategy(Converted from PubMed using Emtree terms)**

#1 'thyroid disease'/exp OR 'autoimmune thyroiditis'/exp OR

'Hashimoto thyroiditis'/exp

#2 'thyroid autoimmunity':ti,ab OR 'thyroglobulin antibody':ti,ab OR

'TgAb':ti,ab OR 'TGAb':ti,ab OR

'thyroid peroxidase antibody':ti,ab OR 'TPOAb':ti,ab OR

'antithyroid antibody':ti,ab

#3 #1 OR #2

#4 'ovarian reserve'/exp OR 'premature ovarian failure'/exp OR

'premature menopause'/exp

#5 'ovarian reserve':ti,ab OR 'diminished ovarian reserve':ti,ab OR

'premature ovarian insufficiency':ti,ab OR

'anti-Mullerian hormone':ti,ab OR 'AMH':ti,ab OR

'antral follicle count':ti,ab

#6 #4 OR #5

#7 #3 AND #6 AND [humans]/lim AND [female]/lim AND [2014-2025]/py

### **3. Chinese Database Search Strategy (CNKI)**

### **Search fields: Title, Keywords, Abstract**

**Search Set 1 (Thyroid autoimmunity):**

Thyroglobulin antibody OR anti-thyroglobulin antibody OR TgAb OR TGAb OR autoimmune thyroid OR Hashimoto thyroiditis OR thyroid peroxidase antibody OR TPOAb OR autoimmune thyroid disease

**Search Set 2 (Ovarian function):**

Ovarian reserve OR diminished ovarian reserve OR Anti-Mullerian Hormone (AMH) OR ovarian insufficiency OR premature ovarian insufficiency OR premature ovarian failure OR antral follicle count (AFC)

**Final search:** Set 1 AND Set 2

**Time range:** January 1, 2014 – January 1, 2025

**Document type:** Journal articles (excluding dissertations and conference abstracts)

### **4. Study Identification Summary**

| Database | Number | Remarks |
| --- | --- | --- |
| PubMed | 6 | Final search date: Jan 1, 2025 |
| Embase | 7 | Jan 1, 2025 |
| Web of Science | 11 | Jan 1, 2025 |
| CNKI | 3 | Jan 1, 2025 |
| Other sources | 6 | Jan 1, 2025 |
| Total (before deduplication) | 33 |  |
| Total (after deduplication) | 30 | Using EndNote |
